# Suppression of prostate tumor cell survival by antisense oligonucleotide-mediated inhibition of *AR-V7* mRNA synthesis

**DOI:** 10.1038/s41388-019-0696-7

**Published:** 2019-01-21

**Authors:** Maria V. Luna Velez, Gerald W. Verhaegh, Frank Smit, J. P. Michiel Sedelaar, Jack A. Schalken

**Affiliations:** 1 Department of Urology, Radboud Institute for Molecular Life Sciences, Radboud university medical center, Nijmegen, The Netherlands; 2 MDxHealth, Nijmegen, The Netherlands

**Keywords:** Targeted therapies, Mechanisms of disease, Prostate cancer

## Abstract

One of the mechanisms by which advanced prostate cancer develops resistance to androgen deprivation therapy is the elevated expression of C-terminally truncated androgen receptor (AR) variants. These variants, such as AR-V7, originate from aberrant splicing of the *AR* pre-mRNA and the inclusion of a cryptic exon containing a premature stop codon in the mRNA. The resulting loss of the ligand-binding domain allows AR-V7 to act as a constitutively active transcription factor. Here, we designed two antisense oligonucleotides (AONs) directed against cryptic splicing signals within the *AR* pre-mRNA. These two AONs, AON-ISE and AON-ESE, demonstrated high efficiency in silencing *AR-V7* splicing without affecting full-length AR expression. The subsequent downregulation of AR-V7-target gene *UBE2C* was accompanied by inhibition of androgen-independent cell proliferation and induction of apoptosis in castration-resistant prostate cancer (CRPC)-derived cell line models 22Rv1, DuCaP, and VCaP. Our results show that splicing-directed AONs can efficiently prevent expression of *AR-V7*, providing an attractive new therapeutic option for the treatment of CRPC.

## Introduction

Despite the clinical remission achieved by androgen deprivation therapy, advanced prostate cancer eventually progresses into recurrent or castration-resistant prostate cancer (CRPC) [1]. CRPC is a lethal disease with no curative treatment available and with a median survival of 1–2 years [2, 3]. One mechanism of CRPC progression is the elevated expression of androgen receptor splice variants (AR-Vs). Although many AR-Vs have been described, *AR*-splice variant 7 (*AR-V7*) is the most commonly and abundantly expressed variant in human CRPC tissues [4, 5]. AR-V7 expression is correlated with a bad prognosis and a high probability of disease recurrence [4–7]. AR-V7 lacks the ligand-binding domain (LBD) and is constitutively active, i.e., it can promote androgen-independent cell proliferation in vitro [8] and tumor growth in vivo under castrate androgen levels [5]. Due to the lack of the LBD, its activity is insensitive to the AR antagonists, bicalutamide and enzalutamide, agents currently used as prostate cancer therapeutics [8–11].

*AR-V7* originates from alternative splicing of the *AR* pre-mRNA. A typical splicing process requires the coordinated action of splicing factors and *cis*-acting regulatory elements. Intron 3 of the *AR* contains two splicing signals known as intronic and exonic splicing enhancers (ISE and ESE, respectively). Recognition of these *cis* elements by the splicing machinery results in the inclusion of a cryptic exon 3 (CE3) into the mRNA. This cryptic exon includes a premature stop codon leading to the synthesis of AR-V7 [12]. Blocking these signals could prevent splicing and inclusion of CE3, leading to the expression of a full-length *AR* mRNA (*AR-FL*) and this potentially could be used as a mean to re-sensitize tumor cells to current androgen deprivation therapy.

Antisense oligonucleotides (AONs) are single stranded, short molecules that can block (aberrant) splicing events by base-pairing with cryptic splice sites in the pre-mRNA in the nucleus [13, 14]. In the present study, we describe the use of AONs to prevent *AR-V7* mRNA synthesis in CRPC-derived cell line models 22Rv1, DuCaP, and VCaP. We show that splicing-directed AONs specifically and efficiently knockdown expression of this variant. The AON-mediated suppression of AR-V7 has an inhibitory effect of androgen-independent cell proliferation. Our results provide the first proof of principle for the use of splice-switching AONs in CRPC and highlights their potential as therapeutic agents.

## Results

### Identification of *cis*-acting splicing enhancer elements in AR CE3

**Fig. 1:**
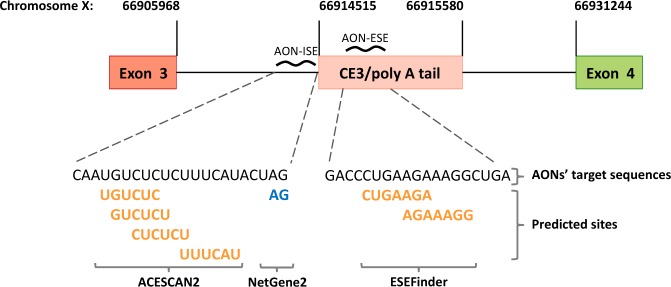
Design of antisense oligonucleotides (AONs). Schematic representation (not to scale) of the AONs designed to prevent splicing of *androgen receptor* (*AR*) pre-mRNA into *AR-V7* mRNA. AON-ISE is complementary to the intronic splicing enhancer (ISE) sites predicted by ACESCAN2, and the cryptic “GA” splice acceptor dinucleotide motif, predicted by NetGene2. AON-ESE is complementary to the region harboring the ESEfinder-predicted exonic splicing enhancer (ESE) sites. Predicted splicing enhancer sites are bold and yellow, and the predicted cryptic splice acceptor site is on blue. The corresponding genomic coordinates (Human Genome Assembly February 2019, HG19) are marked by vertical lines pointing at the 5′ and/or 3′ junctions of exon 3, cryptic exon 3 (CE3), and exon 4

For the identification of *cis-*acting splicing enhancer elements within the so-called CE3 sequence [4, 5] and its flanking regions, we used the publically available computer-based algorithms ACESCAN2 [15] and ESEFinder [16, 17] to predict potential ISE and ESE sites, respectively. Four ISE sites were identified in the flanking region upstream of CE3 and its cryptic splice acceptor (SA) site. This SA site was detected by screening the same sequence with the NetGene2 server [18]. Two ESE sites were found close to the 3′ end of the CE3 sequence. One AON, named AON-ISE, was designed such that it encompasses all four ISE motifs, as well as the detected cryptic SA site (Fig. 1). A second AON, designated AON-ESE, was designed encompassing both ESE motifs in CE3 (Fig. 1). Both AONs were generated with a phosphorothioate backbone [19] and 2′-O-methyl group modifications at the sugar chain [20, 21] to make them resistant to RNAse activity.

### AON-mediated suppression of AR-V7 mRNA synthesis and expression

**Fig. 2:**
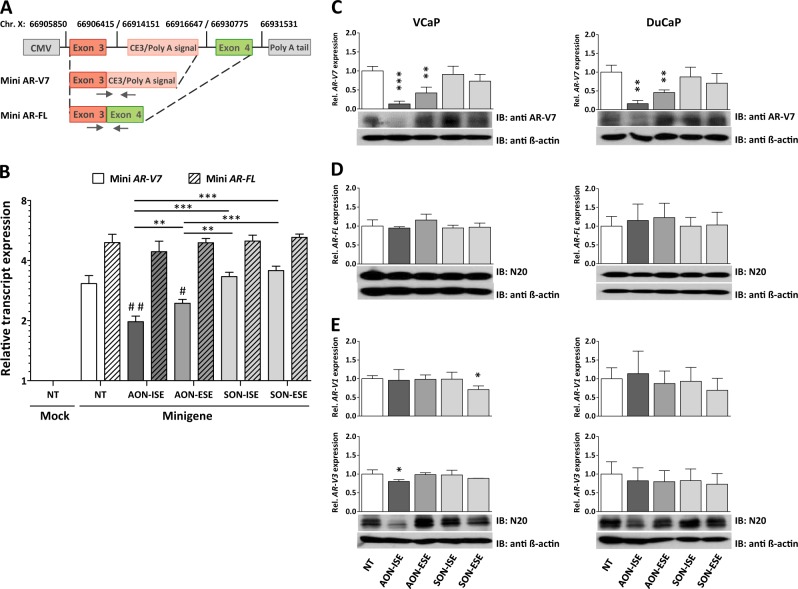
Antisense oligonucleotide (AON)-mediated AR-V7 knockdown. **a** Schematic diagram (not to scale) of the androgen receptor (AR) minigene construct. Minimal regions containing *AR* exon 2, cryptic exon 3 (CE3), exon 4 and their flanking regions are cloned into a CMV-driven pEGFP-N3 expression vector. Vertical lines mark positions of each *AR* gene fragment on chromosome X (Human Genome Assembly February 2019, HG19). Primers for RT-qPCR are marked with headed arrows. **b** AR-negative MIA PaCa-2 cells were transfected with 500 ng AR minigene vector or with empty vector and with 0.5 µM AONs (AON-intronic splicing enhancer (ISE) and AON-exonic splicing enhancer (ESE)) or control sense oligos (SON-ISE and SON-ESE). Relative expression of AR-FL and AR-V7 from the AR minigene were measured by RT-qPCR analysis, four days after transfection. Unpaired *t-*test; **p < 0.01; ***p < 0.001. AON-treated vs non-treated cells; ^#^*p* < 0.05 and ^##^*p* < 0.01. Bars represent the mean ± SD of three independent experiments. **c**–**e** Expression levels of *AR-V7, AR-FL, AR-V1*, and *AR-V3* in DuCaP and VCaP cells (determined by RT-qPCR), 4 days after transfection with 0.2 µM AONs or control oligos. Expression levels were compared with non-transfected cells (NT). Below each graphs, western blot analysis of AR-V7 (anti-AR-V7), AR-FL (N20), and truncated AR-Vs (N20) protein levels are shown. Protein levels of β-actin (anti-β-actin) were used as protein loading control. Unpaired *t*-test; *p < 0.05; **p < 0.01; ***p < 0.001. Bars represent the mean ± SD of three independent experiments

Next, we evaluated the splicing inhibitory potential of the AONs in vitro. An *AR* minigene was created with CE3 and its flanking regions inserted in between exon 3 and exon 4 and flanking regions of the human *AR* gene (Fig. 2a). The *AR* minigene was transiently transfected into AR-negative MIA-PaCa-2 cells (Supplementary Fig. S1A), and both an *AR-FL* (exon 3–exon 4) and an *AR-V7* (exon 3–CE3) transcript were expressed, suggesting that canonical and alternative splicing occurs in the minigene-encoded *AR* transcript (Fig. 2b). Of note, a natural preference for canonical splicing was apparent as levels of the *AR-FL* transcript were almost twofold higher than those of *AR-V7* transcript. Minigene-transfected MIA PaCa-2 cells were subsequently treated with either AON-ISE or AON-ESE. Both splicing-directed AONs displayed a significant reduction of *AR-V7* transcript expression but did not affect the expression levels of *AR-FL* (Fig. 2b). Interestingly, the AON directed against the ESE was less efficient in the knockdown of *AR-V7* than the one directed against the ISE. The specificity of both AONs was assessed by transfecting control oligonucleotides containing the AON sequence in the sense orientation. Neither of the sense oligonucleotides, SON-ISE or SON-ESE, affected the levels of either *AR* minigene-encoded transcript, whereas expression levels were comparable to non-treated minigene-expressing cells (Fig. 2b).

We further tested the AONs ability to knockdown *AR-V7* in the CRPC-derived DuCaP and VCaP cell line models. Both cell lines express *AR-FL* and *AR-V7* at levels comparable to those from CRPC specimens (Supplementary Fig. S1A,B). Upon addition of either AON, a strong decrease in *AR-V7* mRNA expression was noted in both cell lines (Fig. 2c). Western blot analysis of whole-cell extracts using an AR-V7-specific antibody directed against CE3-encoded amino acids showed a reduction of AR-V7 protein levels upon treatment with the AONs (Fig. 2c). Treatment with control SONs did not affect *AR-V7* mRNA or protein levels. *AR-FL* mRNA, as well as protein levels, remained unchanged upon treatment with AONs (Fig. 2d). Because CE3 lies in close proximity to other intronic regions that can serve as cryptic exons to generate other AR variants, such as *AR-V1* or *AR-V3*, we assessed the expression levels of these variants. *AR-V1* mRNA levels were not affected in neither cell line. However, *AR-V3* expression was significantly reduced in VCaP upon addition of AON-ISE, albeit to a lesser extent than that of AR-V7 expression. Staining with an AR N-terminus-specific antibody (N20) detected two protein bands of about 75 kDa. The upper band had the same size as the translated product in AR-V7-transfected HeLa cells (data not shown), suggesting it corresponds to endogenous AR-V7 protein levels (67 kDa). This band was weakened after treatment with AON-ISE, similarly to band detected with AR-V7-specific antibody. Interestingly, the lower molecular size band (~66 kDa) was also reduced in VCaP cells, presumably corresponding to AR-V3 (Fig. 2e). Altogether, these results showed that AONs complementary to the splice enhancer motifs in and around CE3 can efficiently prevent AR-V7 mRNA synthesis in vitro.

### AON-mediated knockdown of AR-V7 results in downregulation of AR-V7-target genes

**Fig. 3:**
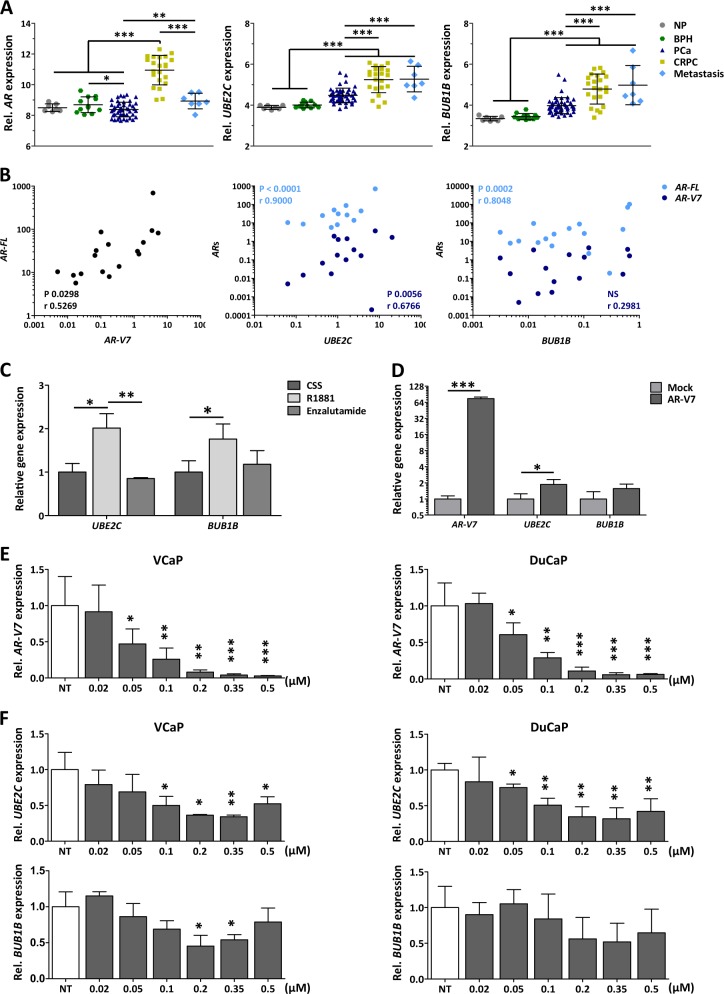
Effect of antisense oligonucleotide (AON)-intronic splicing enhancer (ISE) on androgen receptor (AR)-V7-targeted gene expression. **a** Microarray analysis showing *AR, UBE2C*, and *BUB1B* gene expression profiles in normal prostate (NP, *n* = 7), benign prostate hyperplasia (BPH, *n* = 12), primary prostate cancer (PCa, *n* = 49), castration-resistant prostate cancer (CRPC, *n* = 22), and metastasis (*n* = 7). Microarray values (2 log scale are shown as the mean ± SD of each group is depicted. Unpaired *t*-test; **p < 0.01; ***p < 0.001). **b** Pearson correlation of *AR-FL, AR-V7, UBE2C*, and *BUB1B* mRNA expression, obtained by RT-qPCR, in CRPC (*n* = 20) specimens. Two-tailed *p*-values and Pearson *r* values are depicted. NS; *p* > 0.05. **c** Relative *UBE2C* and *BUB1B* mRNA expression in VCaP cells, determined 96 h after treatment with 0.1 nM R1881, or R1881 in combination with 2 µM enzalutamide. Unpaired *t*-test; *p < 0.05; **p < 0.01; ***p < 0.001. Bars represent the mean ± SD of three independent experiments. **d** Relative mRNA expression levels of *AR-V7, UBE2C*, and *BUB1B* in VCaP cells following transfection with an AR-V7 expression vector. Unpaired *t*-test; *p < 0.05; ***p < 0.001). Bars represent the mean ± SD of three independent experiments. **e, f** Relative mRNA expression from *AR-V7* (e)*, UBE2C* and *BUB1B* (f) in DuCaP and VCaP cells, as determined 96 h after treatment with increasing doses of AON-ISE, compared with non-transfected cells (NT). Unpaired *t*-test; *p < 0.05; **p < 0.01; ***p < 0.001. Bars represent the mean ± SD of three independent experiments

AR-Vs have been described to have an overlapping but distinct transcriptional output than AR-FL [6, 22]. Among the genes described to be regulated by AR-Vs, specifically by AR-V7, are the cell cycle regulatory genes *UBE2C* and *BUB1B* [8, 11]. Microarray analysis of prostate (cancer) specimens showed that, similarly to *AR* expression*, UBE2C* and *BUB1B* are significantly upregulated in CRPC tissue compared with benign tissues and androgen-sensitive primary prostate cancer and metastatic tissues (Fig. 3a). To be able to discriminate between *AR-FL* and *AR-V7* expression, a qPCR validation was performed using CRPC samples from an independent cohort. Both *AR-FL* and *AR-V7* expression positively correlated with the expression of *UBE2C*, but only *AR-FL* correlated with *BUB1B* expression (Fig. 3b).

We next assessed the dependency of *UBE2C* and *BUB1B* expression on AR-FL-mediated transactivation. Treatment of VCaP cells with androgens induced expression of AR-target gene *KLK3* whereas treatment with enzalutamide, a new-generation (full-length) AR antagonist, inhibited it (Supplementary Fig. S2A). Androgen stimulation also resulted in a marked induction of *UBE2C* and *BUB1B*, which was reverted with the addition of enzalutamide (Fig. 3c). Forced expression of *AR-V7* in VCaP cells resulted in a significant upregulation of *UBE2C* and a weak, but observable increase of *BUB1B* expression (Fig. 3d). These last results were obtained from cells grown in androgen-depleted medium, and hence this expression profile was considered a consequence of AR-V7 activity, exclusively. From these results, it is clear that *UBE2C* is part of both AR-FL and AR-V7 transcriptional program and, therefore, it can be used to monitor the efficiency of AON-induced *AR-V7* knockdown.

Because AON-ISE resulted in a more efficient reduction of *AR-V7* expression levels in both DuCaP and VCaP cells than AON-ESE, further experimentation did not include the latter AON. Cells were transfected with various concentrations of AON-ISE ranging from 0.02 µM to 0.5 µM. A significant *AR-V7* knockdown was achieved with doses above 0.02 µM, with a clear dose-dependent decrease of *AR-V7* mRNA levels in both cell lines (Fig. 3e). The dose-dependent AON-ISE-mediated knockdown of *AR-V7* resulted in a dose-dependent suppression of *UBE2C*. Although the maximum level of *AR-V7* splicing inhibition was achieved at 0.5 µM AON-ISE, a dose of 0.35 µM suppressed *UBE2C* the most. Treatment with AON-ISE at a dose of 0.2 µM and 0.35 µM doses resulted in a significant downregulation of *BUB1B* in VCaP cells, but the AON did not affect *BUB1B* expression in DuCaP cells (Fig. 3f). AON-ISE treatment resulted in a specific *AR-V7* knockdown and subsequently downregulation of the AR-V7-target gene, *UBE2C*.

Intra-chromosomal translocation of the transmembrane protease serine 2 (*TMPRSS2*) gene to the ETS family member *ERG* is the most prevalent fusion in prostate cancer [23] and the fusion gene is expressed in the VCaP cell line. AR-V7, as well as AR-FL, have been described to mediate transcriptional activation of *TMPRSS2* [4, 12, 24]. Interestingly, under castrated conditions, AON-ISE treatment of VCaP cells resulted in downregulation of *TMPRSS2-ERG* mRNA levels (Supplementary Fig. S2B), suggesting the involvement of AR-V7 in the transcriptional regulation of this fusion gene.

### Effects of AON-ISE-mediated AR-V7 knockdown on cell proliferation and apoptosis

**Fig. 4:**
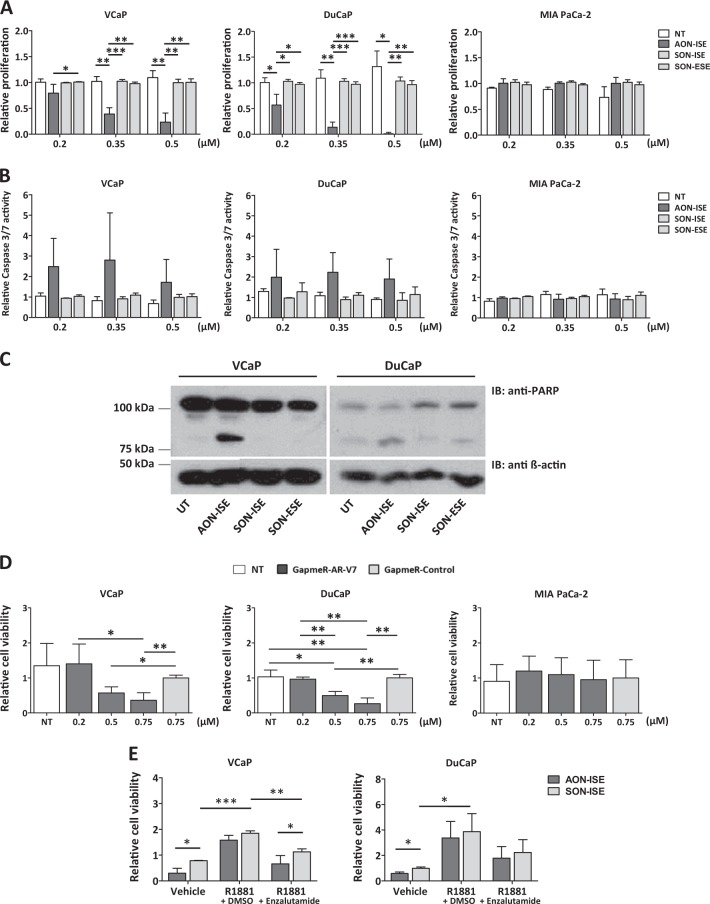
Effect of antisense oligonucleotide (AON)-intronic splicing enhancer (ISE) in cell viability and apoptosis. **a** Dose-dependent effect of AON-ISE-mediated androgen receptor (AR)-V7 knockdown on cell viability of DuCaP and VCaP cells compared with SON-treated cells. AR-negative MIA PaCa-2 cells were used as a negative control. Unpaired *t*-test; *p < 0.05; **p < 0.01; ***p < 0.001. Bars represent the mean ± SD of three independent experiments. **b** Induction of apoptosis, as determined by Caspase-3/7 induction, in DuCaP and VCaP cells after treatment with different doses of AON-ISE. AR-negative MIA PaCa-2 cells were used as a negative control. Bars represent the mean ± SD of three independent experiments. **c** Western blot analysis of full-length and cleaved PARP-1 protein (anti-PARP) in DuCaP and VCaP cells, 96 h after transfection with 0.2 µM AON-ISE or sense oligonucleotides. Protein levels of β-actin (anti-β-actin) was used as loading control. **d** Relative cell viability of DuCaP, VCaP, and MIA PaCa-2 cells after treatment with increasing doses of GapmeR-AR-V7, compared with GapmeR-Control-treated cells. Unpaired *t-*test; *p < 0.05; **p < 0.01. Bars represent the mean ± SD of three independent experiments. **e** Relative cell viability of AON-ISE or SON-ISE-transfected (0.2 µM) DuCaP and VCaP cells grown in medium containing 0.1 nM R1881, or R1881 in combination with 2 µM enzalutamide. Unpaired *t*-test; *p < 0.05; **p < 0.01; ***p < 0.001. Bars represent the mean ± SD of three independent experiments

Prostate cancer cells rely on androgens for proliferation and survival, via activation of AR-FL and its targeted genes. One of the functional consequences of AR-V7 protein expression is its capacity to maintain proliferation of tumor cells in the absence of androgens. Thus, we evaluated the ability of AON-ISE to inhibit androgen-independent cell proliferation. To eliminate any contribution of AR-FL, cells were grown in androgen-depleted medium. A dose-dependent effect on cell viability was observed in DuCaP and VCaP upon treatment with AON-ISE but not with control SONs (Fig. 4a). To exclude that the effect of AON-ISE on cell viability is AR independent, we assessed the effect of the AON on cell viability of AR-negative MIA PaCa-2 cells. Treatment with three different concentrations of AON-ISE had no effect on MIA PaCa-2 cell viability (Fig. 4a). The reduction of cell viability was found to be a result of the induction of apoptosis, which was marked by an increase in Caspase-3/7 activity (Fig. 4b) and, consequently, a cleavage of the Poly (ADP-ribose) polymerase 1 (PARP-1) protein [25, 26] (Fig. 4c).

A GapmeR AON was designed to bind complementary to *AR-V7* mRNA, inducing its degradation by RNAse H (Supplementary Fig. S3 and Methods). GapmeR treatment caused a reduction in cell viability of both DuCaP and VCaP cells similarly to the AON-ISE treatment, without affecting MIA PaCa-2 cell viability (Fig. 4d). This validated the association of *AR-V7* knockdown with the decrease in cell viability.

Reactivation of full-length AR signaling was expected to revert the cell survival inhibitory effect of the AR-V7-targeting AONs. Upon stimulation of DuCaP or VCaP cells with the synthetic androgen R1881, AR-FL activity became re-activated (Supplementary Fig. S2A) and cell viability was not significantly affected by AON-ISE compared with control SON-ISE-treated cells. Addition of enzalutamide to the medium re-sensitized cells to AON-ISE treatment (Fig. 4e), demonstrating that AON-ISE is able to inhibit AR-V7-mediated and androgen-independent induction of cell proliferation.

Finally, to validate the effects of AON-ISE in a cell line with a different genetic background, the CRPC-derived 22Rv1 cell line was used. 22Rv1 cells express AR-V7 at a similar level than DuCaP and VCaP cells, and the ratio of *AR-V7*-to-*AR-FL* mRNA levels in 22Rv1 cells was higher than in these two cell lines, making it an ideal model to study AR-V7 activity (Supplementary Fig. S1A,B). AON-ISE treatment reduced cell viability of 22Rv1 cells by induction of apoptosis at all doses tested (Supplementary Fig. S4A,B). In addition, *AR-V7* and *UBE2C* but not *AR-FL* mRNA levels were significantly downregulated upon treatment with AON-ISE (Supplementary Fig. S4C).

### Effect of AON-ISE treatment over time

**Fig. 5:**
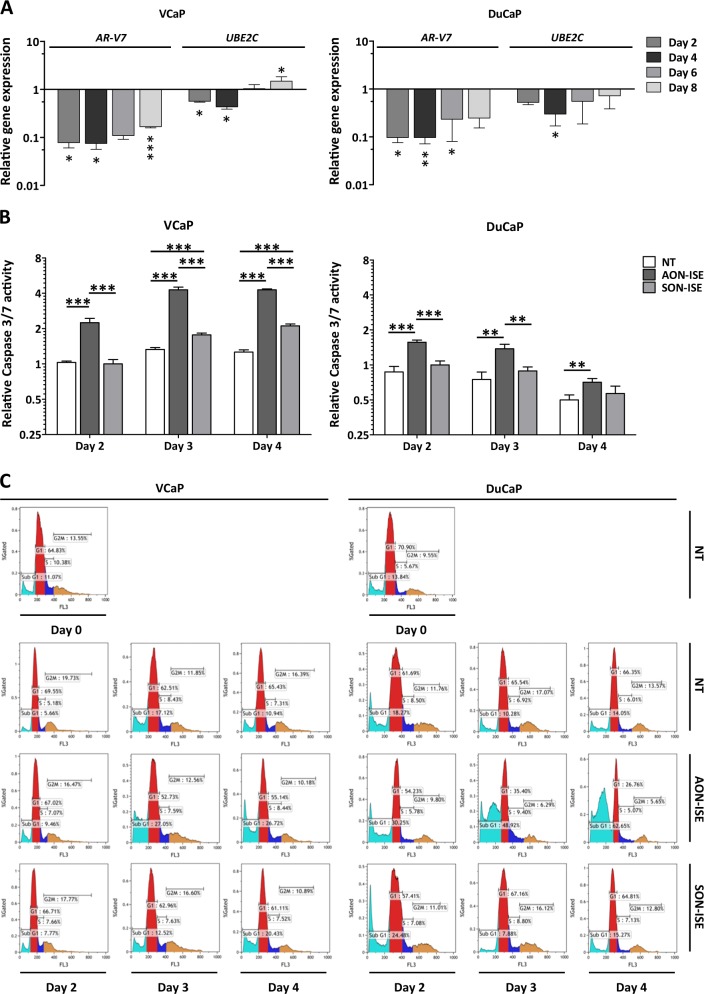
Time follow-up of antisense oligonucleotide (AON)-intronic splicing enhancer's (ISE’s) effect. **a** Expression levels of *AR-V7* and *UBE2C* in DuCaP and VCaP cells, determined at different time points after treatment with 0.2 µM of AON-ISE, normalized to values from un-transfected cells. Unpaired *t-*test; *p < 0.05; **p < 0.01; ***p < 0.001. Bars represent the mean ± SD of three independent experiments. **b** Relative Caspase-3/7 activity measured at different time points in DuCaP and VCaP cells treated with 0.2 µM of AON-ISE or SON-ISE. Unpaired *t*-test; **p < 0.01; ***p < 0.001. Bars represent the mean ± SD of three independent experiments. **c** Increase in DuCaP and VCaP sub-G1 cell population at day 3 and day 4, following knockdown of *AR-V7* by AON-ISE, as analyzed by propidium iodide staining and flow cytometry. Graphs show gated percentages of cells corresponding to sub-G1 (sky blue), G1 (red), S (blue), and G2/M (yellow) cell cycle phases

To assess whether the effect on cell viability matches the downregulation of *UBE2C*, apoptosis and gene expression were assessed in time following AON-ISE treatment. A robust downregulation of *AR-V7* was observed in both DuCaP and VCaP cells at all time points up to 8 days after a single administration of the AON. The highest knockdown efficiency was observed between day 2 and day 4 hours after transfection with a slow decrease in knockdown thereafter. Consistently, expression levels of *UBE2C* followed a similar trend (Fig. 5a).

Caspase-3/7 induction was observed from day 2 onwards in both CRPC cell lines after treatment with AON-ISE (Fig. 5b). Cell cycle profiling of these cells showed an increase in the sub-G1 cell population in AON-ISE-treated cells at day 3 after transfection (Fig. 5c). While the number of apoptotic cells in VCaP remained stable over time, an increase was noted in DuCaP cell cultures (Fig. 5c). Results in MIA PaCa-2 cells showed no significant difference in the induction of caspase activity or the number of (sub-G1) apoptotic cells between AON and SON-treated cells (Supplementary Fig. S5).

## Discussion

Therapeutic drugs for advanced PCa are designed to block androgen signaling, either by interfering with the androgen synthesis or by blocking AR androgen binding. Androgen deprivation therapy in patients with advanced or metastasized disease gives a clear survival benefit of about 18 months, but ultimately tumor cells become resistant leading to CRPC. While AR-targeting drugs efficiently target the full-length receptor, they have no effect on C-terminally truncated AR variants that increase upon CRPC development. Several AR variants, such as AR-V7, act as constitutively active transcription factors and promote androgen-independent cell proliferation. Here, we have described a proof of principle study where we employed AON technology to prevent splicing of CE3 of *AR-V7*. These AONs reduced AR-V7 mRNA and protein levels, without affecting full-length AR mRNA and protein, and induced apoptosis of CRPC cells in a low androgen environment.

The publically available computer-based algorithms ACESCAN2 and ESEFinder were used to predict potential splicing enhancers within CE3 and its flanking regions. An ISE and an ESE site in and around CE3 of the *AR* pre-mRNA have been reported. Mutations in these motifs impaired binding of splicing factors U2AF65 and ASF/SF2 and prevented the generation of an *AR-V7* transcript [12]. In our results, several ISE and ESE sites were predicted in and around CE3, and all (partially) overlapped with the ISE and ESE motifs described before [12]. This suggests that not a single ISE or ESE site may be pinpointed as most relevant for *AR-V7* mRNA splicing, and hence, AON-ISE and AON-ESE were designed to encompass all predicted ISE and ESE motifs, respectively. Comparing both AONs used in this study, AON-ESE affected the generation of *AR-V7* transcripts at a much lesser extent than AON-ISE. The AON-ISE sequence is directed not only against the predicted ISE motifs but also against the cryptic SA site. The fact that SA sites are essential for splicing, and enhancers only stimulate the splicing process, may explain why AON-ISE is more effective in blocking *AR-V7* splicing than AON-ESE. Furthermore, in the context of the AR transcript, the ISE enhancer may have superior activity over the ESE enhancer, as has been suggested by Liu et al. [12].

The use of a minigene was key to confirm the disturbance in splicing and to validate the importance of the predicted ISE and ESE sites. Upon transfection, both *AR-FL* and *AR-V7* transcripts were expressed from the minigene construct. Interestingly, relative expression of the *AR-V7* transcript from the minigene was lower than that of *AR-FL*. Although this might suggest an intrinsic preference of splicing factors for binding to canonical splicing signals, this effect could as well be linked to the particular pool of splicing factors present in the AR-negative MIA PaCa-2 cell line and therefore expression levels of the two mini transcripts could differ among cell line models.

Both of our AONs were generated with a phosphorothioate backbone and a 2′-O-methyl group modification at the sugar chain, chemical modifications that make them resistant to RNAse activity. This could allow our AONs to remain stable throughout turnover of pre-existing AR-V7 proteins achieving a maximum effect. The physiological effect of AON-ISE occurred between day 3 and day 4, likely the time required for degradation of AR-V7 protein to a threshold that rendered cells sensitive to the oligo treatment.

Curiously, AON-ISE treatment in VCaP cells resulted in the downregulation of another AR mRNA variant, *AR-V3*. The intronic region where CE3 and the predicted ISE sites lie, also harbors the cryptic exon that is present in the *AR-V3* splice variant [4, 27]. AON-ISE binding to its target sequence could disturb recruitment of splicing factors to one or more unknown splicing signals critical for the synthesis of *AR-V3* mRNA. Although AR-V3 is one of the most abundant variants, AR-V3 is considered inactive due to its permanent cytoplasmic localization, and therefore functional consequences of *AR-V3* knockdown are unlikely.

AON treatment demonstrated efficacy in three CRPC cell line models, but the response of each cell line to the treatment was not identical. At the mRNA and protein level, the effect of AON treatment seemed to be less pronounced in DuCaP than in VCaP cells. This probably reflects the difference in *AR* transcript levels between these two cell lines. *AR-FL*, as well as *AR-V7* mRNA expression, is markedly higher in DuCaP than in VCaP, resulting from a higher *AR* copy number (Supplementary Fig. S1C). This also could explain why the effect of AON-ISE on *AR-V3* mRNA levels was only visible in VCaP cells, but not in DuCaP. Contradictory to the knockdown efficiency, the effect of AON-ISE in cell viability of DuCaP was a lot more striking than in VCaP cells. DuCaP cells showed a stronger dose-dependent sensitivity to AON-ISE treatment with a higher cleaved-to-full-length PARP-1 ratio and a consistent higher amount of apoptotic cells than the counterpart cell line. These observations indicated that the physiological dependency on AR-V7 for cell survival or proliferation, but not the knockdown efficiency per se dictates the sensitivity to AON-ISE treatment.

It has been shown recently that degradation products of AONs can affect cell proliferation and differentiation [28]. Sense oligonucleotides (SONs) having identical backbone modifications as the AONs were used in our experiments as negative controls. None of the SONs affected cell viability of the transfected cells, even at the highest dose tested (0.5 µM), highlighting the specificity of our system. Furthermore, treatment with AON-ISE in the AR-negative MIA PaCa-2 showed no signs of AON-mediated toxicity at all concentrations tested.

Androgen stimulation has been reported to reduce *AR* mRNA levels by repressing *AR* transcription, a process that can be reverted upon treatment with AR inhibitors or following androgen withdrawal [29, 30]. In VCaP cells, this negative feedback was evident by a reduction in the expression levels of *AR-FL* and *AR-V7* mRNA upon treatment with R1881. Transcripts levels were restored after addition of enzalutamide to the culturing medium. Here, androgen stimulation, as well as overexpression of *AR-V7*, resulted in a significant induction of *UBE2C* whereas enzalutamide treatment or AON-ISE-mediated knockdown of *AR-V7* promoted its downregulation, validating it as both an *AR-FL*- and an *AR-V7*-target gene.

Another gene reported to be regulated by both AR-FL and AR-V7 is *TMPRSS2* [4, 12, 24]. Fusion of the *TMPRSS2* and *ERG* genes is the most prevalent gene fusion in prostate cancer, occurring in about 50% the cases [23]. Knockdown of *TMPRSS2-ERG* has been reported to inhibit cell proliferation in vitro [31], and knockdown of *ERG* reduced tumor progression in vivo in an orthotopic mouse model [32]. AON-ISE-mediated *AR-V7* knockdown resulted in downregulation of *TMPRSS2-ERG* mRNA levels in VCaP cells, arguing for a role of AR-V7 in the maintenance of cell viability via *TMPRSS2-ERG* activation. The effect of AON-ISE in cell viability, however, was also observed in 22Rv1 cells (*TMPRSS2-ERG* fusion gene negative). Thus, AR-V7-mediated cell viability cannot be solely attributed to the regulation of *TMPRSS2-ERG*.

Previous studies have shown that *UBE2C* knockdown downregulates cell proliferation and activates the cellular apoptosis pathway [33]. AR-V7 has been reported to mediate *UBE2C* transcriptional activation by direct occupancy of its promoter [24]. Interestingly, AON-ISE-mediated knockdown of *AR-V7* and the subsequent downregulation of *UBE2C* were accompanied by an increase in Caspase-3/7 activity and an inhibition of androgen-independent cell proliferation. This suggests a role for *UBE2C* as a key regulator of AR-V7-promoted cell viability.

Sustained cell proliferation in CRPC cells under castrate levels of androgens clearly demands for the development of new AR-targeted therapies. AONs have been shown to mediate significant biological effects in vitro and in vivo, reverting diverse disease phenotypes [34–37]. AON technology is nearing clinical relevance as it offers novel therapeutic opportunities for severe, untreatable or chronic diseases, including cancer [38–43]. Here, we present the first report on the use of a splice-switching AON technology in CRPC. Our results showed an AON-mediated specific and efficient knockdown of *AR-V7*, which induces cell death in three CRPC-derived cell line models. These results certainly are promising and warrant further in vivo investigation.

Targeting of internal organs like the prostate or disseminated prostate cancer cells, typical for CRPC, would require systemic administration of AONs. Systemic administration of splice-switching AONs has demonstrated significant clinical benefits before. AON treatment corrected the open reading frame of the dystrophin *DMD* mRNA and ameliorated symptoms in Duchenne muscular dystrophy patients [44, 45]. Pharmacokinetic and pharmacodynamic profiling of our AONs is pivotal for further optimization of our AON as a drug system [46, 47]. The AON chemistry design and the choice of delivery method will ultimately determine its targeting efficacy.

## Materials and methods

### Splicing signals prediction

A 4-kb sequence containing an *AR* intronic region known as CE3 and its flanking sequences (516-bp upstream and 2418-bp downstream of CE3) was screened for the presence of intronic and exonic splice enhancer motifs. The publically available computer-based algorithms ACESCAN2 (http://genes.mit.edu/ acescan2/index.html) and ESEFinder (http://rulai.cshl.edu/cgi-bin/tools/ESE3/esefinder.cgi) were used to predict potential ISEs and ESEs, respectively. The cryptic splicing acceptor site was detected by screening the same sequence with the NetGene2 server (http://www.cbs.dtu.dk/services/NetGene2/).

### Design of AONs

Two RNA AONs, a 22 nucleotides long AON-ISE and a 19 nucleotides long AON-ESE, together with two control sense RNA oligonucleotides SON-ISE and SON-ESE were synthesized and modified with a phosphorothioate backbone and 2′-O-methyl groups at the sugar chain (Eurogentec, The Netherlands). Oligos were dissolved in nuclease-free water. A 20 nucleotides long GapmeR AONs, GapmeR-AR-V7, was designed using SFold software [48] (http://sfold.wadsworth.org/cgi-bin/index.pl). The chimeric GapmeR (RNA5-DNA10-RNA5) was chemically modified and synthesized as described above for the RNA AONs. The GapmeR sequence described by Wheeler et al. [49] was used as a control. Analytical ion exchange high-pressure liquid chromatography (HPLC) and matrix-assisted laser desorption/ionization time-of-flight mass spectrometer (MALDI-TOF MS) were chosen to assess the purity of all oligonucleotides, and a purity of >90% was considered as pure. AON sequences are listed in Supplementary Table S1.

### Cell culture

The CRPC-derived 22Rv1 (ATCC# CRL-2505), DuCaP, and VCaP (kindly provided by dr. Kenneth J. Pienta, Johns Hopkins, Baltimore, USA), the prostate cancer-derived LNCaP (ATCC# CRL-1740), PC-3 (ATCC# CRL-1435), and the bladder carcinoma-derived 5637 (ATCC# HTB-9) cell lines were maintained as monolayer cultures in Roswell Park Memorial Institute (RPMI)-1640 medium (Invitrogen), supplemented with 2 mM l-glutamine and 10% fetal calf serum (FCS; Sigma). Pancreatic carcinoma MIA-PaCa-2 cells (ATCC# CRL-1420) were grown in Dulbecco’s modified Eagle's medium (DMEM) (Invitrogen) with 4.5 g/ml glucose and 1 mM pyruvate, supplemented with 2 mM l-glutamine and 10% FCS and 2.5% of horse serum (Invitrogen). For hormone stimulation experiments, 0.1 nM of synthetic androgen R1881 (PerkinElmer) was added to the medium in combination with 2 µM enzalutamide (Selleck Chemicals) or 0.2% dimethyl sulfoxide (as a vehicle control). Results were reproduced in at least three independent experiments. All cultures were maintained in a humidified atmosphere at 37 °C and 5% CO_2_. Cell lines were authenticated in 2016 using the PowerPlex 21 system (Promega) by Eurofins Genomics (Germany). Cells were frequently tested for *Mycoplasma* infection, using a *Mycoplasma*-specific PCR, and propagated for no >6 months or 30 passages after resuscitation from the authenticated stocks.

### Construction of minigene and AR-V7 expression vector

The AR minigene was built according to sequence coordinates described by Liu et al. [12]. Briefly, three PCR amplicons were generated using Phusion High-Fidelity DNA Polymerase (New England Biolabs) and joined together by SOEing PCR. Genomic DNA from the (normal) human genomic DNA was used as the template to amplify AR exon 3, CE3, exon 4, and their flanking regions. Exon 4 was amplified including a downstream 447-base-pair flanking region. CE3 was amplified including 364-bp upstream and 1067-bp downstream flanking regions and *AR* exon 4 amplicon included 469 bp from the upstream flanking region. For SOEing PCR, all three fragments contain 20-bp overlapping sequences incorporated as overhangs in the forward and reverse primers. The assembled minigene was directionally cloned into the pEGFP-N3 vector (Clontech) between the *Bgl*II and *Not*I sites (thereby removing the eGFP region). For cloning of the eukaryotic expression vector pCMV-AR-V7, CE3 was amplified from human genomic DNA. The fusion between an exon 3 and CE3 was achieved by SOEing PCR using Phusion High-Fidelity DNA Polymerase (New England Biolabs). The forward primers for the sewed amplicons were complemented with a 5′-GAGATG-3′ overhang and a *Hin*dIII site, and the reverse primers with a 5′-GTTGTT-3′ following an *Mfe*I restriction site. The insert was directionally cloned into the pEGFP-N3-derived CMV-driven expression vector backbone vector. Correct cloning was verified by Sanger DNA sequence analysis of PCR products, purified using Wizard PCR preps DNA purification system (Promega). Primer sequences for cloning and sequencing analysis are listed in Supplementary Table S2.

### Transfection with AONs

One day before transfection, 140,000 cells (DuCaP/VCaP) or 70,000 cells (22Rv1/MIA-PaCa-2) were seeded per well of a 24-well plate, in a total volume of 500 µl medium. After trypsinization, cells were collected and seeded in charcoal-stripped serum-containing medium to wash away traces of androgens previously reported to be present in FCS [50]. Transfection mixtures were prepared by combining oligonucleotides (AON/SON or GapmeRs) in a desired concentration with X-tremeGENE™ 9 transfection reagent (Roche), both dissolved in Opti-MEM I Reduced serum-free medium (Invitrogen). A mix of transfection reagent alone, i.e., without oligonucleotide, was used as non-transfected control. Mixes were incubated at room temperature for 15 min before addition to the cells in a dropwise manner. For overexpression studies, 140,000 VCaP cells were seeded per well in 24-well plates. Twenty-four hours later, cells were transfected with 250 ng of pCMV-AR-V7 expression vector or empty vector control. For minigene experiments, 70,000 MIA-PaCa-2 cells were seeded per well in 24-well plates and after 24 h, cells were co-transfected with 500 ng of minigene or empty vector and 0.5 µM of the desired oligonucleotide. All experiments were performed at least three times.

### RNA isolation and reverse transcription-PCR

Total RNA was isolated using TRIzol reagent (Invitrogen) according to manufacturer’s protocol. Concentration and purity of the RNA was determined on a Nanodrop-1000 spectrophotometer (Thermo Scientific). Subsequently, 2 μg of total RNA was treated with DNaseI and used to synthesize complementary DNA using random hexamer primers and SuperScript II Reverse Transcriptase (Invitrogen). Real-time PCR (qPCR) analysis was performed using LightCycler 480 SYBR Green I Master Mix (Roche) and gene-specific primers (Supplementary Table S3). Crossing-point (Cp) values were determined using the LightCycler 480 SW 1.5 software (Roche). RNA not subjected to reverse transcriptase was used as a control for non-specific PCR amplification. Expression levels of the human heterochromatin protein 1 binding protein 3 (*HP1BP3*), the hypoxanthine phosphoribosyltransferase 1 (*HPRT1*) and the glyceraldehyde-3-phosphate dehydrogenase (*GAPDH*) genes were used for normalization and relative gene expression levels were calculated according to the mathematical model for relative quantification in real-time PCR [51]. To determine *TMPRSS2-ERG* fusion transcript levels, a forward primer directed to exon 1 of the *TMPRSS2* transcript together with a reverse primer directed to exon 4 of the *ERG* transcript were used [52].

### Western blot analysis

One day before transfection, 1,200,000 (DuCaP/VCaP) cells were seeded in 10-cm dishes. When cells reached 70% confluency, oligonucleotide transfection was performed. Four days after transfection, cells were harvested and washed. Cell pellets were lysed using Laemmli lysis buffer (1 mM CaCl_2_, 2% sodium dodecyl sulfate (SDS), 60 mM Tris-Glycine pH 6.8) supplemented with 1:50 β-mercaptoethanol (Merck). Lysates were homogenized by sheering them through a 0.5 × 25 mm syringe needle. Protein concentration was measured using the Odyssey CLx Imaging System (LI-COR) and Image Studio software (LI-COR), after staining with Coomassie brilliant blue (Merck) with serial dilutions of bovine serum albumin as a standard. A total of 100 µg of protein was subjected to SDS-polyacrylamide gel electrophoresis using 7.5% polyacrylamide gels. Proteins were electrotransferred onto polyvinylidene fluoride membranes (Hybond 0.45 µm, Amersham Biosciences). Membranes were blocked for 1 h in phosphate-buffered saline with Tween-20 (PBS-T)/5% non-fat dry milk and incubated overnight with the primary antibody. The mouse monoclonal-antibody anti-AR-V7 (Precision Antibody, #AG10008), the rabbit polyclonal AR antibody N20 (Santa Cruz, SC-816), the rabbit monoclonal-antibody anti-PARP (Cell Signaling, #46D11), and the mouse monoclonal-antibody anti-β-actin (Sigma-Aldrich, clone AC-15) were used, diluted 1:500, 1:50,000, 1:1000, and 1:5000 in PBS-T/5% non-fat dry milk, respectively. The Horseradish peroxidase-conjugated donkey-anti-rabbit antibody (Amersham Biosciences, N4934) or sheep-anti-mouse antibody (Amersham Biosciences, NXA931) diluted 1:50,000 in PBS-T were used as secondary antibodies. Protein bands were detected using ECL and Hyperfilm (Amersham Biosciences). Results were reproduced in two independent experiments.

### Tissue collection and processing

CRPC tissue (*n* = 20) was obtained by transurethral resection of prostate tumor tissue (TURP). TURP specimens were snap frozen in liquid nitrogen. Regions of CRPC tumors (*n* = 20) with high percentage of epithelial tumor cells (>50%) were selected for cryo-sectioning. The use of patient materials was approved by the local ethics committee of the Radboud university medical center (CMO Arnhem-Nijmegen).

### Microarray analysis

Microarray gene expression analysis on normal prostate, prostate cancer, and CRPC tissue samples were performed and described previously by Leyten et al. [53].

### Cell viability assay

To assess cell viability, 10,000 (22Rv1), 20,000 cells (DuCaP/VCaP), or 500 cells (MIA-PaCa-2) were cultured in 96-well culture plates. Transfection with oligonucleotides was done 24 h after seeding. Four days after transfection, cell viability was measured using 3-(4,5-dimethylthiazol-2-yl)−2,5-dephenyltetrazolium bromide (MTT, 1 mg/ml) assays. Alternatively, cell viability was measured using CellTiter-Glo luminescence assays (Promega), following the manufacturer’s instructions. Absorbance (at 490 nm) and luminescence were measured using a Victor3 multilabel reader (PerkinElmer). Medium only was used as background control. To calculate the relative cell viability, cell viability values for each condition were normalized to the average of the cell viability values for control oligo-transfected cells. Each experiment was performed in triplicate and repeated at least three times.

### Apoptosis assay

In parallel to the cell viability assays, cells were seeded into 96-well plates for assessment of Caspase-3/7 activity using the Apo-ONE Homogenous Caspase-3/7 Assay (Promega), following manufacturer’s instructions. After 4 h of incubation, luminescence was measured on a Victor3 multilabel reader (PerkinElmer). The luminescence signal from medium alone was used as background. Caspase-3/7 activity was normalized to values in control oligo-transfected cells. Each experiment was performed in triplicate and repeated at least three times.

### Cell cycle analysis

One day before transfection, 280,000 cells (DuCaP/VCaP) were seeded per well of a 12-well plate. The next day, cells were transfected with oligonucleotides as described above. Samples were harvested at different time points after transfection. Briefly, cells were harvested and washed with 0.9% NaCl. Cell pellets were resuspended in Hank's balanced salt solution (Invitrogen) and cells were fixed with ice-cold ethanol (58%). Fixated cells were centrifuged, resuspended in PBS, and treated with RNase A (100 µg/ml, Sigma) for 40 min at 37 °C. Subsequently, cells were stained with propidium iodide (40 µg/ml, Sigma) for 15 min in the dark. The samples were analyzed on a FC500 Flow Cytometer (Beckman-Coulter) and histograms were created and analyzed using Kaluza® Flow Analysis software (Beckman-Coulter). Results were reproduced in two independent experiments.

### Statistical analysis

The data are presented as means ± SD from at least three independent experiments. Two-tailed paired and unpaired *t*-tests were performed using GraphPad Prism (GraphPad Software, Inc.). Pearson correlation coefficients were used to determine the relationships between relative gene expression profiles, considering a 95% confidence interval. A *p*-value of < 0.05 was considered statistically significant and *p* < 0.05 is represented by one star (*), *p* < 0.01 is represented by two stars (**), and *p* < 0.001 is represented by three stars (***).

## Supplementary information

Supplementary Tables

AR mRNA expression and AR copy number in CRPC tissue and in cell line models

AR-FL and AR-V7 signaling

GapmeR-mediated knockdown of AR-V7

Effect of AON-ISE-mediated AR-V7 knockdown in 22Rv1 cells

Assessment of cell death in MIA PaCa-2

Supplementary Figures and Tables Legends
