# Supplementary material for: Suppression of prostate tumor cell survival by antisense oligonucleotide-mediated inhibition of AR-V7 mRNA synthesis
Source: Oncogene. 2019 Jan 21;38(19):3696–709. doi: 10.1038/s41388-019-0696-7 (PMC6756119; doi:10.1038/s41388-019-0696-7)
Supplement: Supplementary file 1 — Supplementary Tables [file 41388_2019_696_MOESM1_ESM.docx]

**Supplementary tables**

Supplementary Table 1. Oligonucleotide sequences.

| **AONs** | **5’ 🡪 3’** |
| --- | --- |
| AON-ISE | mC*mU*mA*mG*mU*mA*mU*mG*mA*mA*mA*mG*mA*mG*mA*mG*mA*mC*mA*mU*mU*mG |
| AON-ESE | mU*mC*mA*mG*mC*mC*mU*mU*mU*mC*mU*mU*mC*mA*mG*mG*mG*mU*mC |
| **SONs** | **5’ 🡪 3’** |
| SON-ISE | mC*mA*mA*mU*mG*mU*mC*mU*mC*mU*mC*mU*mU*mU*mC*mA*mU*mA*mC*mU*mA*mG |
| SON-ESE | mG*mA*mC*mC*mC*mU*mG*mA*mA*mG*mA*mA*mA*mG*mG*mC*mU*mG*mA |
| **GapmeRs** | **5’ 🡪 3’** |
| GapmeR-AR-V7 | mG*mU*mC*mA*mU*T*T*T*G*A*G*A*T*G*C*mU*mU*mG*mC*mA |
| GapmeR-Control | mC*mC*mU*mU*mC*C*C*T*G*A*A*G*G*T*T*mC*mC*mU*mC*mC |

m = 2’O-Me RNA; * = phosphorothioate backbone

Supplementary Table 2. Primer sequences for cloning of the AR minigene and AR-V7 expression vector.

| **Cloning minigene** |  | **Forward (5’ 🡪 3‘)** | **Reverse (5’ 🡪 3‘)** | |
| --- | --- | --- | --- | --- |
| PCR#1 | | TCGTTGAGATCTAGGGAAACAGAAGTACCTGT | GAAGAGAAGGGGGATATTCCCAGGTGTTCA | |
| PCR#2 | | GGAATATCCCCCTTCTCTTCTCCTACATCTTCTC | TGGACTGGAAAATGGAGATAGGTGAGGAAGAAC | |
| PCR#3 | | TATCTCCATTTTCCAGTCCAACGTTTTCTA | TTGTTGGCGGCCGCCAGGCAAGGCCTTGGCC | |
| PCR#4 | | TCGTTGAGATCTAGGGAAACAGAAGTACCTGT | TGGACTGGAAAATGGAGATAGGTGAGGAAGAAC | |
| PCR#5 | | TCGTTGAGATCTAGGGAAACAGAAGTACCTGT | TTGTTGGCGGCCGCCAGGCAAGGCCTTGGCC | |
| **Cloning  pCMV-AR-V7** |  | **Forward (5’ 🡪 3‘)** | **Reverse (5’ 🡪 3‘)** | |
| PCR#1 | | GAGATGAAGCTTCTGGGTGTCACTATG | CGGAATTTTTCTCCCAGAGTCATCCCTGCT | |
| PCR#2 | | GGGAGAAAAATTCCGGGTTGGCAAT | TTGTTGCAATTGTCAGCAATCAAGAGAGTAG | |
| PCR#3 | | GAGATGAAGCTTCTGGGTGTCACTATG | TTGTTGCAATTGTCAGCAATCAAGAGAGTAG | |
| **Sanger Sequencing** |  | **(5’ 🡪 3‘)** | | |
| hAR-Intron2-F | | TCGTTGAGATCTAGGGAAACAGAAGTACCTGT | |  |
| hAR-Intron3-R | | GAAGAGAAGGGGGATATTCCCAGGTGTTCA | |  |
| hAR-Intron3-F | | GGAATATCCCCCTTCTCTTCTCCTACATCTTCTC | |  |
| hAR-CE3-F | | TATCTCCATTTTCCAGTCCAACGTTTTCTA | |  |
| hAR-CE3-R | | TGGACTGGAAAATGGAGATAGGTGAGGAAGAAC | |  |
| hAR-Exon4-R | | TTGTTGGCGGCCGCCAGGCAAGGCCTTGGCC | |  |
| hAR-Intron3-F2 | | GGAAGAAACTTTGCTGATGGG | |  |
| hAR-Intron3-F3 | | GGAGAAGTGGAGTCTGTGAAGC | |  |
| hAR-DBD-F | | GGTGAGCAGAGTGCCCTATC | |  |

Supplementary Table 3. Primer sequences for (real-time) PCR analysis.

| **RT-PCR** |  | **Forward (5’ 🡪 3’)** | | **Reverse (5’ 🡪 3’)** |
| --- | --- | --- | --- | --- |
| *AR-FL* |  | AAGGAACTCGATCGTATCATTGC | TTGGGCACTTGCACAGAGAT | |
| *AR-V7* |  | CGTCTTCGGAAATGTTATGAAGC | GAATGAGGCAAGTCAGCCTTTCT | |
| *UBE2C* |  | AGTGGCTACCCTTACAATGCG | TCAAGGGACTATCAATGTTGGG | |
| *BUB1B* |  | CTTCTCAACCTGTCCCATGG | CTGCTTTGTGTAGCATCTCCAC | |
| *KLK3* |  | CAGCTGCCCACTGCATCAGG | GGGTGAACTTGCGCACACAC | |
| *AR-FL (minigene)* |  | ATCTTGTCGTCTTCGGAAATGT | AAGCCTCTCCTTCCTCCTGTAG | |
| *AR-V7 (minigene)* |  | CAGGGATGACTCTGGGAGAA | AGCCCTCTAGAGCCCTCATTT | |
| *HPRT1* |  | CTCAACTTTAACTGGAAAGAATGTC | TCCTTTTCACCAGCAAGCT | |
| *HP1BP3* |  | TGGAATATGCAATCTTGTCTGC | GAACCCTTTCCCAGAGATCTG | |
| *TMPRSS2-ERG* |  | CGCGAGCTAAGCAGGAG | GTCCATAGTCGCTGGAGGAG | |
| *GAPDH* |  | TCAAGGCTGAGAACGGGAAG | TGGACTCCACGACGTACTCA | |
| **gDNA PCR** |  | **Forward (5’ 🡪 3’)** | **Reverse (5’ 🡪 3’)** | |
| *GAPDH* |  | CCCACACACATGCACTTACC | TTGCCTGTCCTTCCTAGCTC | |
| *AR* |  | CGACCAGATGGCTGTCATTC | CTGGAGTTGACATTGGTGAAGG | |
| *SPIN4* |  | TTCGAGCGTTTCTGCCAAAA | TGCGATCTCAAAGACCTGGT | |
