# Supplementary figures and images for: Suppression of prostate tumor cell survival by antisense oligonucleotide-mediated inhibition of AR-V7 mRNA synthesis
Source: Oncogene. 2019 Jan 21;38(19):3696–709. doi: 10.1038/s41388-019-0696-7 (PMC6756119; doi:10.1038/s41388-019-0696-7)

## Slide 1
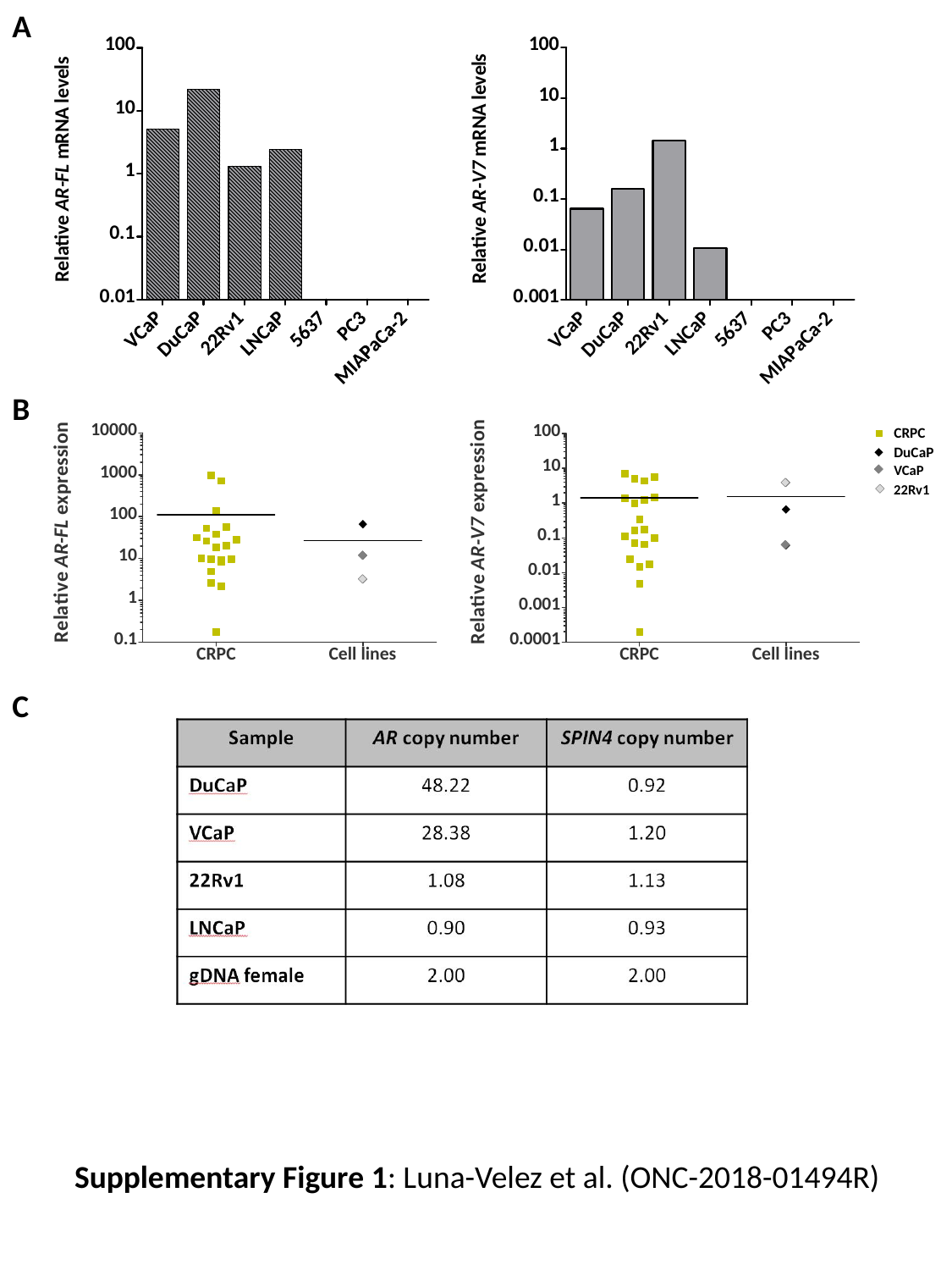

A
B
VCaP
22Rv1
C
Supplementary Figure 1: Luna-Velez et al. (ONC-2018-01494R)

Supplement: Supplementary file 2 — AR mRNA expression and AR copy number in CRPC tissue and in cell line models [file 41388_2019_696_MOESM2_ESM.pptx]

## Slide 1
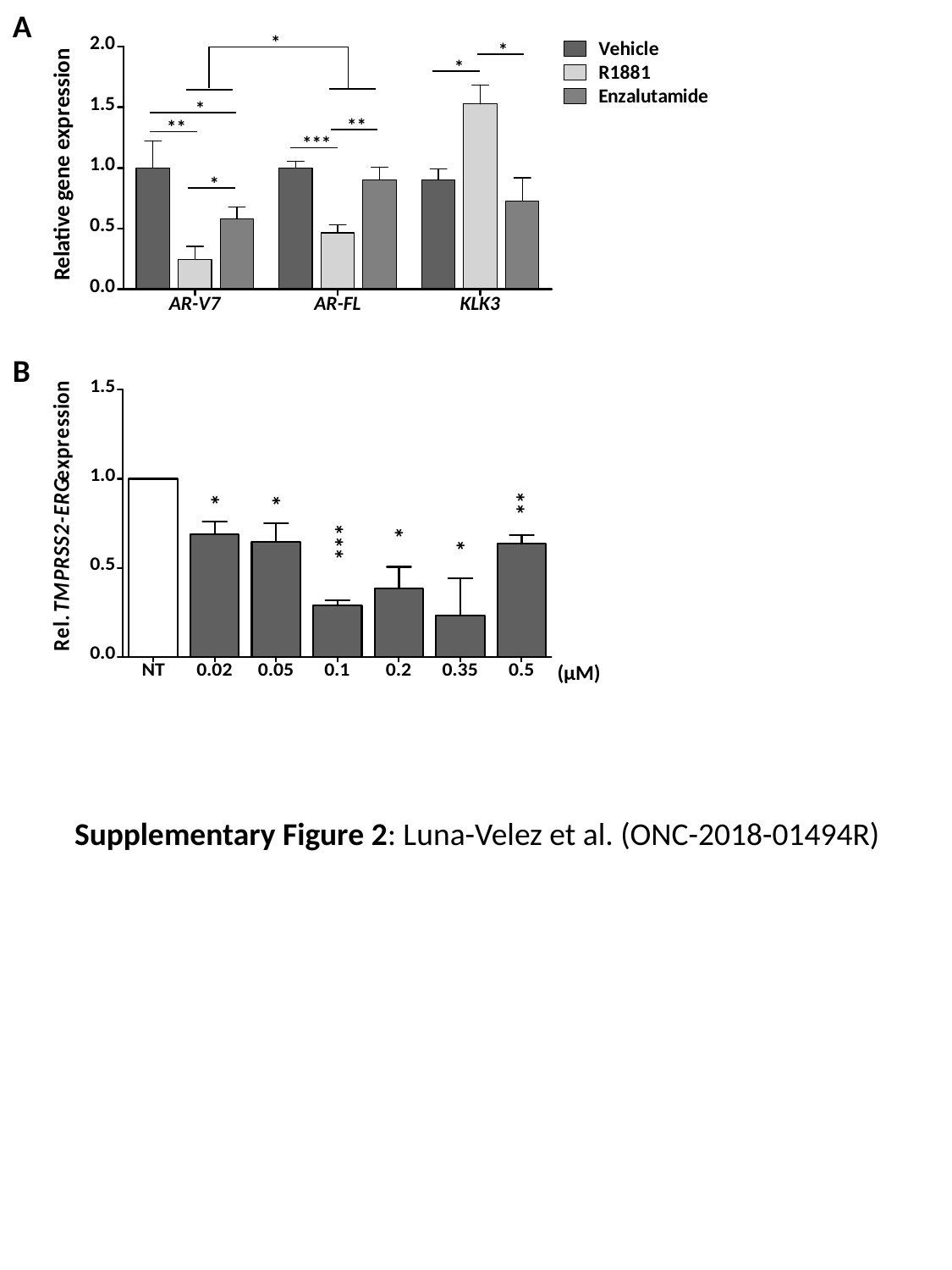

A
*
*
*
*
**
**
***
*
B
*
*
**
*
***
*
(µM)
Supplementary Figure 2: Luna-Velez et al. (ONC-2018-01494R)

Supplement: Supplementary file 3 — AR-FL and AR-V7 signaling [file 41388_2019_696_MOESM3_ESM.pptx]

## Slide 1
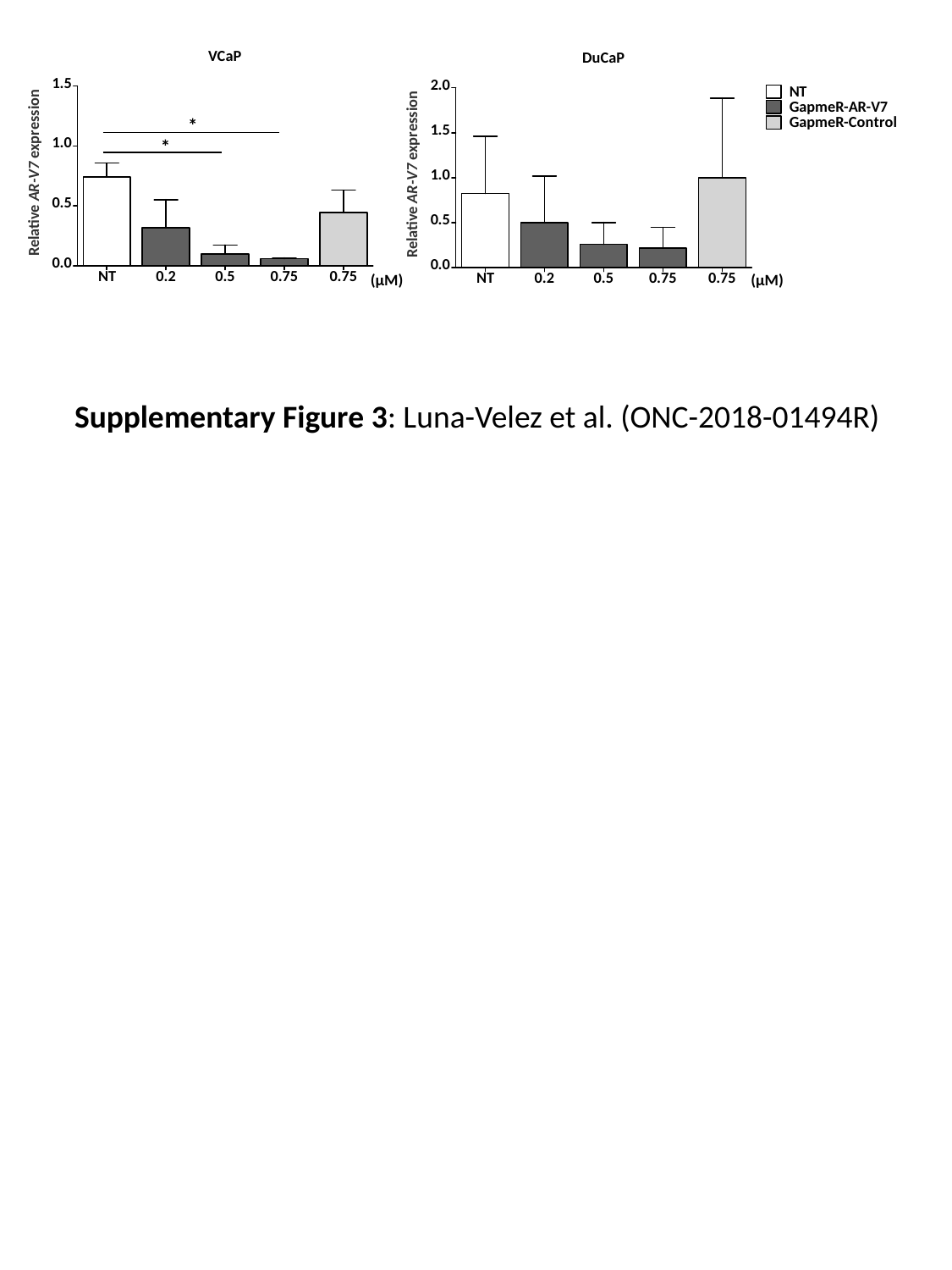

*
*
(µM)
(µM)
Supplementary Figure 3: Luna-Velez et al. (ONC-2018-01494R)

Supplement: Supplementary file 4 — GapmeR-mediated knockdown of AR-V7 [file 41388_2019_696_MOESM4_ESM.pptx]

## Slide 1
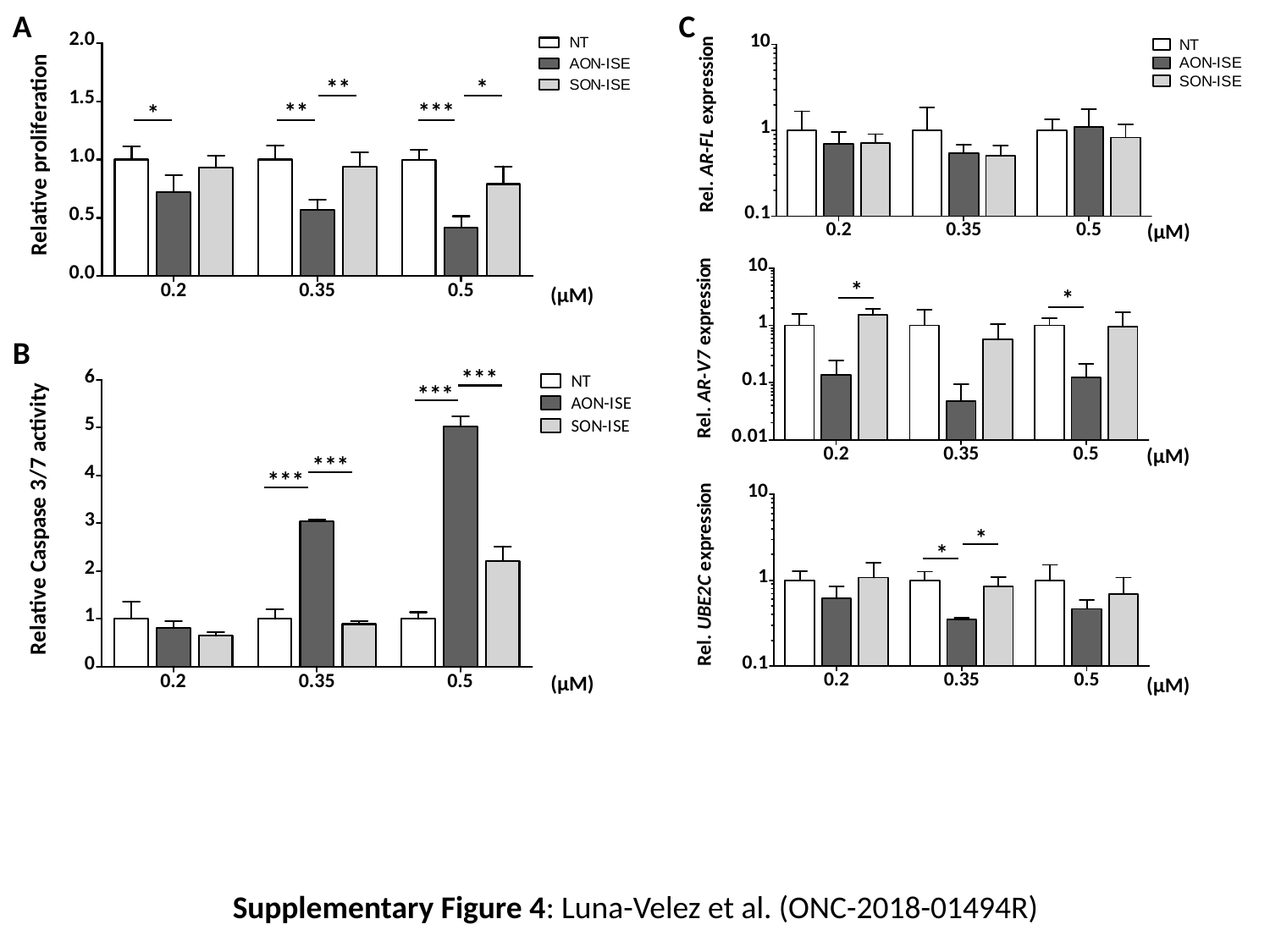

A
C
**
*
**
***
*
(µM)
(µM)
*
*
(µM)
*
*
(µM)
B
***
***
***
***
(µM)
Supplementary Figure 4: Luna-Velez et al. (ONC-2018-01494R)

Supplement: Supplementary file 5 — Effect of AON-ISE-mediated AR-V7 knockdown in 22Rv1 cells [file 41388_2019_696_MOESM5_ESM.pptx]

## Slide 1
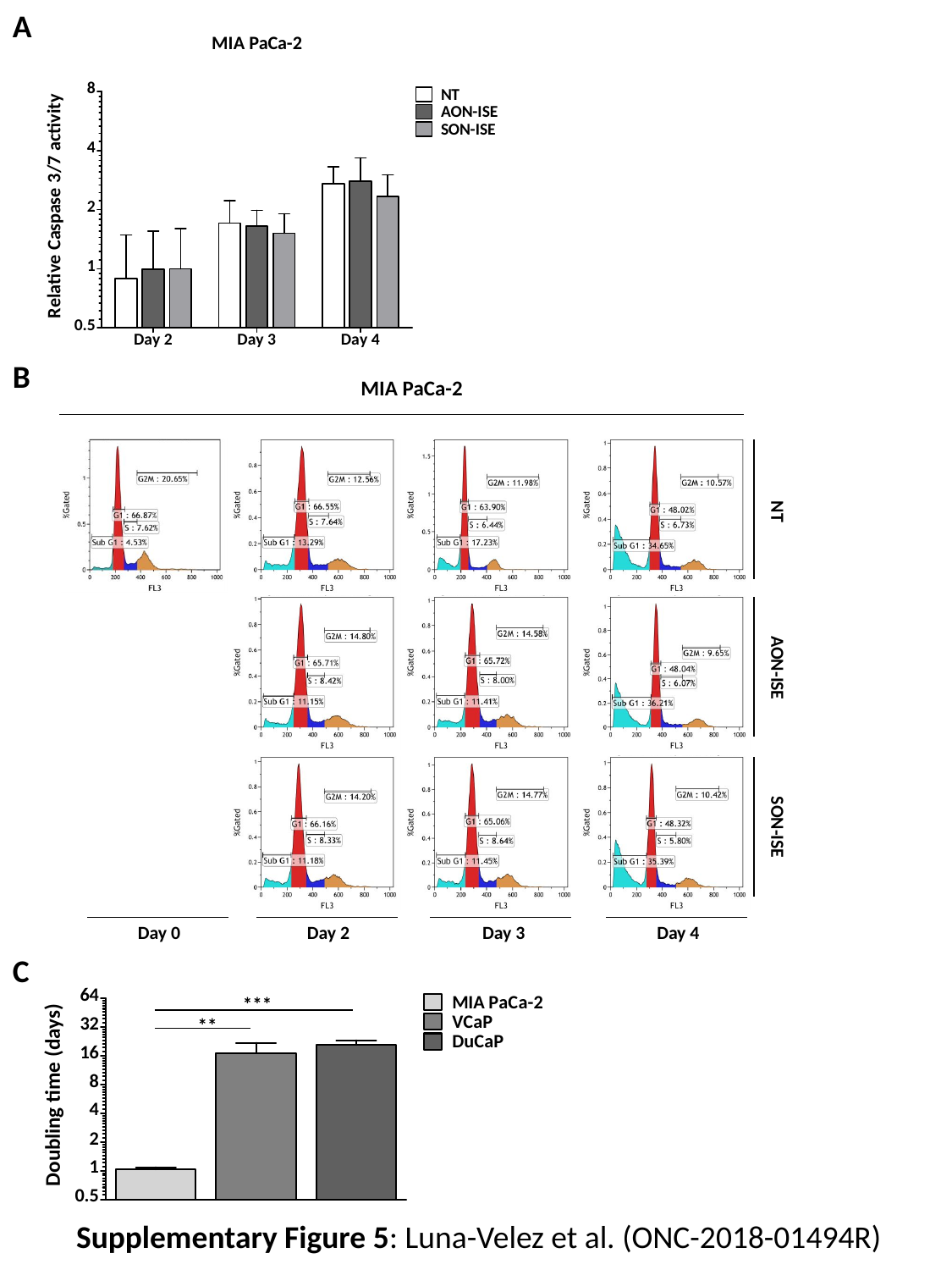

A
B
MIA PaCa-2
NT
AON-ISE
SON-ISE
Day 0
Day 2
Day 3
Day 4
C
***
**
Supplementary Figure 5: Luna-Velez et al. (ONC-2018-01494R)

Supplement: Supplementary file 6 — Assessment of cell death in MIA PaCa-2 [file 41388_2019_696_MOESM6_ESM.pptx]
