# Supplementary material for: Suppression of prostate tumor cell survival by antisense oligonucleotide-mediated inhibition of AR-V7 mRNA synthesis
Source: Oncogene. 2019 Jan 21;38(19):3696–709. doi: 10.1038/s41388-019-0696-7 (PMC6756119; doi:10.1038/s41388-019-0696-7)
Supplement: Supplementary file 7 — Supplementary Figures and Tables Legends [file 41388_2019_696_MOESM7_ESM.docx]

**Supplementary Figure and Table Legends**

**Supplementary Figure Legends**

**Supplementary Figure 1. *AR* mRNA expression and *AR* copy number in CRPC tissue and in cell line models.** (**A**) Relative mRNA expression of *AR-FL* and *AR-V7* in AR-positive prostate cancer cell lines 22Rv1, DuCaP, LNCaP and VCaP, and AR-negative cell lines 5637, MIA PaCa-2 and PC3, as determined by real-time RT-PCR. AR expression levels were normalized to the expression of the *HP1BP3* housekeeping gene. (**B**) Expression levels of *AR-FL* and *AR-V7* in castration-resistant prostate cancer (CRPC, n=20) specimens and in CRPC-derived cell lines 22Rv1, DuCaP and VCaP as a reference. Bars represent the mean of each group. (**C**) *AR* and *SPIN4* gene copy numbers were determined by real-time PCR using genomic DNA from 22Rv1, DuCaP, LNCaP and VCaP cells, and white blood cells from a healthy female. PCR values of the X-linked genes were normalized to PCR values of the autosomal GAPDH gene, and then normalized to the ratio found in female cells.

**Supplementary Figure 2. AR-FL and AR-V7 signaling.** (**A**) Relative *AR-V7, AR-FL and KLK3* mRNA expression in VCaP cells, determined 96 hours after treatment with 0.1 nM R1881, or R1881 in combination with 2 µM Enzalutamide. Unpaired t-test; *, p < 0.05; **, p < 0.01; ***, p < 0.001. Bars represent the mean ± SD of three independent experiments. (**B**) Relative mRNA expression levels of *TMPRSS2-ERG* in VCaP cells following treatment with increasing doses of AON-ISE, compared to non-transfected cells (NT). Unpaired t-test; *, p<0.05; **, p<0.01; ***, p<0.001. Bars represent the mean ± SD of three independent experiments.

**Supplementary Figure 3. GapmeR-mediated knockdown of *AR-V7*.** Relative expression of *AR-V7* in DuCaP and VCaP cells after treatment with control GapmeR or increasing doses of AR-V7 GapmeR. AR-V7 expression levels were normalized to *HP1BP3*. Unpaired t-test; *, p < 0.05. Bars represent the mean ± SD of three independent experiments.

**Supplementary Figure 4. Effect of AON-ISE-mediated AR-V7 knockdown in 22Rv1 cells.** (**A**) Dose-dependent effect of AON-ISE-mediated AR-V7 knockdown on cell viability of 22Rv1 cells compared to non-transfected cells (NT). Unpaired t-test; *, p<0.05; **, p<0.01; ***, p<0.001. Bars represent the mean ± SD of three independent experiments. (**B**) Induction of apoptosis, as determined by Caspase 3/7 induction, in 22Rv1 cells after treatment with different doses of AON-ISE. Unpaired t-test; ***, p<0.001. Bars represent the mean ± SD of three independent experiments. (**C**) Relative mRNA expression levels of *AR-FL, AR-V7* and *UBE2C* in 22Rv1 cells following treatment with increasing doses of AON-ISE, compared to controls. Unpaired t-test; *, p<0.05. Bars represent the mean ± SD of three independent experiments.

**Supplementary Figure 5. Assessment of cell death in MIA PaCa-2.** (**A**) Relative Caspase 3/7 activity measured at different time points in cells treated with 0.2 µM of AON-ISE or SON-ISE, and of non-transfected (NT) cells as a reference. Bars represent the mean ±SD of three independent experiments. (**B**) Cell cycle analysis of MIA PaCa-2 cells after treatment with AON-ISE and SON-ISE, assessed on day 0, 2, 3 and 4 after transfection. Ethanol-fixated and RNase-treated cells were stained with propidium and analyzed by flow cytometry. Percentages of cells in each phase of the cell cycle were determined using the [Kaluza® Flow Analysis software](https://www.beckmancoulter.com/wsrportal/wsrportal.portal?_nfpb=true&_windowLabel=UCM_RENDERER&_urlType=render&wlpUCM_RENDERER_path=%2Fwsr%2Fresearch-and-discovery%2Fproducts-and-services%2Fflow-cytometry%2Fsoftware%2Fkaluza-analysis-software%2Findex.htm), and are depicted in the graphs. (**C**) Cell doubling times were calculated by entering cell viability values, determined using CellTiterGLO, at different time points into the publically available Doubling Time calculator (http://www.doubling-time.com/compute.php). Unpaired t-test; **, p < 0.01; ***, p < 0.001. Bars represent the mean ±SD of three independent measurements.

**Supplementary Table Legends**

Supplementary Table 1. Oligonucleotide sequences. Antisense and sense oligonucleotides, RNA-based and GapmeR (RNA and DNA chimeric molecule). RNA molecule has a 2’O-Me modification and a phosphorothioate backbone.

Supplementary Table 2. Primer sequences for cloning of the AR minigene and AR-V7 expression vector. Primer sequences for SOEing PCR, the cloning strategy for the generation of the AR minigene and the AR-V7 expression vector. Primers for sequencing analysis of synthesized PCR products and generated constructs are included.

Supplementary Table 3. Primer sequences for (real-time) PCR analysis. Primer sequences for (real-time) PCR analysis performed after RNA isolation and reverse transcriptase reaction using diverse prostate specimens.
